# Seco-B-Ring Steroidal Dienynes with Aromatic D Ring: Design, Synthesis and Biological Evaluation

**DOI:** 10.3390/ijms18102162

**Published:** 2017-10-17

**Authors:** Marcin Szybinski, Pawel Brzeminski, Adrian Fabisiak, Klaudia Berkowska, Ewa Marcinkowska, Rafal R. Sicinski

**Affiliations:** 1 Department of Chemistry, University of Warsaw, Pasteura 1, 02-093 Warsaw, Poland; marcin.szybinski@gmail.com (M.S.); pbrzeminski@chem.uw.edu.pl (P.B.); afabisiak@chem.uw.edu.pl (A.F.); 2 Department of Biotechnology, University of Wroclaw, Joliot-Curie 14a, 50-383 Wroclaw, Poland; klaudia.berkowska@uwr.edu.pl (K.B.); ema@cs.uni.wroc.pl (E.M.)

**Keywords:** steroidal dienynes, B-seco steroids, Sonogashira reaction, vitamin D receptor, HL-60 cell differentiation

## Abstract

Continuing our structure-activity studies on the vitamin D analogs with the altered intercyclic seco-B-ring fragment, we designed compounds possessing dienyne system conjugated with the benzene D ring. Analysis of the literature data and the docking experiments seemed to indicate that the target compounds could mimic the ligands with a good affinity to the vitamin D receptor (VDR). Multi-step synthesis of the C/D-ring building block of the tetralone structure was achieved and its enol triflate was coupled with the known A-ring fragments, possessing conjugated enyne moiety, using Sonogashira protocol. The structures of the final products were confirmed by NMR, UV and mass spectroscopy. Their binding affinities for the full-length human VDR were determined and it was established that compound substituted at C-2 with exomethylene group showed significant binding to the receptor. This analog was also able to induce monocytic differentiation of HL-60 cells.

## 1. Introduction

**Figure 1:**
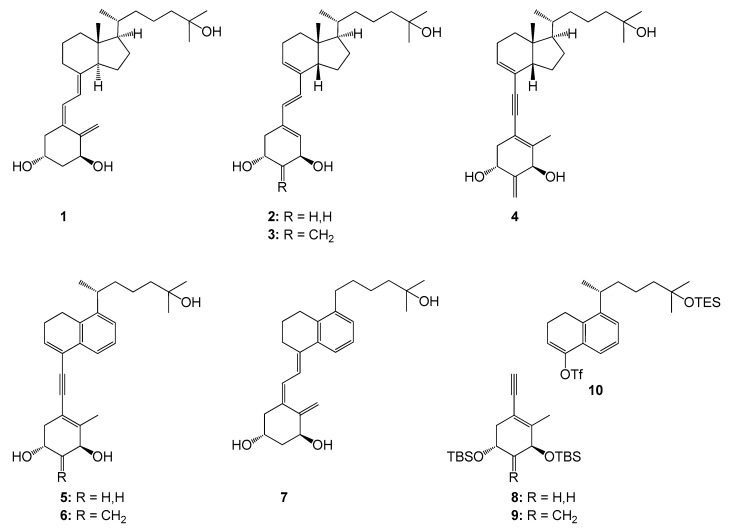
Chemical structure of 1α,25-dihydroxyvitamin D_3_ (**1**), its analogs, and the building blocks for the synthesis of compounds **5** and **6**.

Calcitriol (**1**; 1α,25-dihydroxyvitamin D_3_; 1α,25-(OH)_2_D_3_; Figure 1) is the most active metabolite of the vitamin D_3_ [1], representing its hormonal form [2]. Numerous studies demonstrated that this active form of vitamin D_3_ is responsible for calcium and phosphorus homeostasis, and, additionally, plays an important role in cell proliferation and differentiation as well as apoptosis and immunomodulation [3,4,5]. These activities are primarily mediated through the vitamin D receptor (VDR) [6], a member of a nuclear receptor superfamily [7,8] acting as a ligand-activated transcription factor. It has been established that calcitriol binds to the VDR, and then heterodimerizes with retinoid X receptor (RXR). Upon recruitments of specific coactivators, the formed complex influences the target genes by binding to vitamin D receptor elements (VDRE) [9,10]. Antiproliferative and prodifferentiating effects of calcitriol on various types of malignant cells could be used for the treatment of cancer [11]. However, usefulness of calcitriol as a cancer chemopreventive agent is significantly limited by its strong calcemic effect that could result in hypercalcemia, mineralization of the internal organs, blood vessels and cutaneous tissues. This fact has stimulated broad synthetic efforts in the pharmaceutical companies and academic institutions directed to the development of calcitriol analogs characterized by clear dissociation between the antiproliferative and calcemic activities [12,13,14].

Skeletal modifications in the synthesized vitamin D compounds focused mainly on their A ring and steroidal side chain at C-17. Structural changes in the triene system were significantly less common and involved, for example, hydrogenation of C(10)=C(19) bond [15,16], inverted configuration of C(5)=C(6) [17] or C(7)=C(8) [18,19,20] bond and deletion of an exomethylene unit at C-10 [21,22]. The intercyclic 5,7-diene fragment was modified by its substitution at C-6 [23,24,25,26,27,28,29,30,31,32] and C-7 [32,33]. In 2011, an interesting study was published by Kittaka et al. [34], indicating that more drastic seco-B-ring modification can still result in compounds of potent VDR binding affinity. Thus, the Kittaka group described that 14-epi-19-nortachysterol derivative **2** has a binding ability to the hVDR decreased only seven times compared to that of calcitriol; its 2-methylene substituted analog **3** was even more active. Interestingly, also in the case of compound **4** with the conjugated dienyne fragment, only two-fold reduction of the binding activity has been reported relative to the natural hormone **1** [34].

Taking the above-described facts into consideration, we have decided to broaden the structure-activity studies in this area and synthesize similar seco-B steroidal compounds **5** and **6** characterized by a presence of an aromatic D ring. It should be added that the first report on a new class of vitamin D analogs with a benzene D ring was published by Mouriño et al. in 2010 [35]. However, the synthesized compound **7** slowly equilibrated into a more stable previtamin form. Obviously such isomerization could not occur in our designed compounds **5** and **6**, and their polyunsaturated system, located in all steroidal rings, should be stable. We performed molecular studies with docking these target compounds into the ligand binding domain of the hVDR and both ligands **5** and **6** seemed to show all expected, favorable interactions with the respective amino acids (vide infra).

## 2. Results and Discussion

### 2.1. Chemistry

**Scheme 1:**
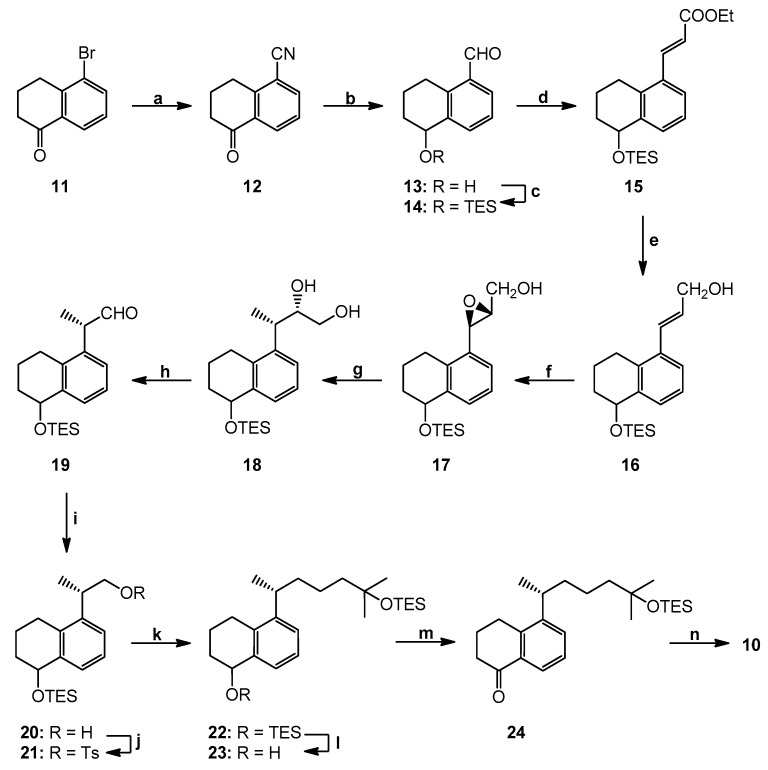
Synthetic route to the vinyl triflate **10**. Reagents and conditions: (**a**) Zn(CN)_2_, Pd(Ph_3_)_4_, *N,N-*dimethylformamide (DMF), 110 °C, 64%; (**b**) diisobutylaluminum hydride (DIBALH), CH_2_Cl_2_, −78 °C, 60%; (**c**) triethylsilyl trifluoromethanesulfonate (TfOTES), 2,6-lutidine, CH_2_Cl_2_, 99%; (**d**) ethyl (triphenylphosphoranylidene)acetate, CH_2_Cl_2_, 0–25 °C, 94%; (**e**) DIBALH, CH_2_Cl_2_, −78 °C, 98%; (**f**) (−)-diisopropyl *D*-tartrate (*D*-(-)-DIPT), Ti(O-*i*Pr)_4_, *tert*-butyl hydroperoxide (TBHP), MS 4Å, CH_2_Cl_2_, 79%; (**g**) CuCN, MeLi, Et_2_O, −78 °C, 80%; (**h**) NaIO_4_, MeOH, 94%; (**i**) NaBH_4_, MeOH, 0–25 °C, 90%; (**j**) *p-*TsCl, triethylamine (TEA), 4-(dimethylamino)pyridine (DMAP), CH_2_Cl_2_, 91%; (**k**) ClCH_2_CH_2_C(CH_3_)_2_OTES (**A**), Mg, CuI, tetrahydrofuran (THF), 72%; (**l**) tetrabutylammonium fluoride (TBAF), THF, 93%; (**m**) Dess–Martin periodinane, CH_2_Cl_2_, 69%; (**n**) *n*-BuLi, diisopropylamine (DIPA), PhNTf_2_, THF, 94%.

We considered Sonogashira coupling of the known A-ring fragments **8** and **9**, prepared in the Norman’s and our laboratory [36,37], with the C/D-ring triflate **10** as a convenient route to the target compounds **5** and **6**. As a starting material served commercially available 5-bromotetralone (**11**; Scheme 1), this was first converted to the corresponding cyano compound **12** using the procedure described by Tschaen et al. [38]. The cyanation method involving the use of CuCN and FeCl_3_ [39] proved to be less efficient. Diisobutylaluminum hydride reduction of **12** gave the racemic hydroxy aldehyde **13**, and its further synthetic transformations were executed on mixtures of compounds being epimeric at C-5.

Silylation of **13** and the following Horner–Wadsworth–Emmons reaction of the protected hydroxy aldehyde **14** with ethyl (triphenylphosphoranylidene)acetate furnished the unsaturated ester **15**. The observed value (15.8 Hz) of the vinylic protons coupling in its ^1^H NMR spectrum proved *E*-configuration of the newly introduced double bond. Reduction of the ester **15** provided the allylic alcohol **16** that was then subjected to Sharpless asymmetric epoxidation using the (−)-diisopropyl *D*-tartrate to generate the epoxide **17** of the desired configuration [40]. Regioselective ring opening of this epoxide with lithium methylcyanocuprate afforded the mono-protected triol **18**. Its subsequent cleavage with sodium periodate gave the aldehyde **19** that was reduced with sodium borohydride to the monoprotected diol **20**. This compound was tosylated and the formed tosylate **21** subjected to alkylation with the Grignard reagent, generated from the chloro ether **A**. This process was carried out in the presence of CuI and resulted in the formation of the product **23** with the desired steroidal side chain. Subsequent oxidation of the hydroxyl group with Dess-Martin periodinane afforded the tetralone derivative **24**. Its enolate form, generated with lithium diisopropylamide, was treated with *N*-phenyltrifluoromethanesulfonimide and the resulting enol triflate **10** was a building block suitable for the planned coupling with the acetylenic compounds **8** and **9**.

**Scheme 2:**
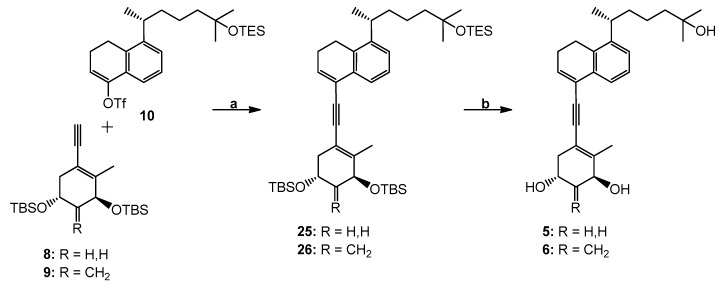
Synthetic route to the target compounds **5** and **6**. Reagents and conditions: (**a**) CuI, (Ph_3_P)_2_Pd(OAc)_2_, diethylamine (DEA), DMF (**25**: 26%, **26**: 23%); (**b**) TBAF, THF (**5**: 85%, **6**: 85%).

Sonogashira reaction of the synthesized enol triflate **10** with the corresponding A-ring fragments **8** and **9** provided the expected dienyne products **25** and **26**, albeit in rather low yield (Scheme 2). Despite our attempts, the outcome of this process could not be improved. Final desilylation of the protected compounds **25** and **26** afforded the target compounds **5** and **6**. All spectroscopic data fully supported the structures of the synthesized compounds.

### 2.2. Docking Studies

The docking simulations of the synthesized compounds **5** and **6** to the ligand binding domain (LBD) of the VDR were performed using Molegro Virtual Docker (release 4.0, CLC bio, Qiagen, Aarhus, Denmark). The LBD was extracted from crystalline hVDR (LBD)-**1** complex (PDB Code: 1DB1) [41]. After docking the energy-minimized structures of the ligands **5** and **6** into VDR, we carefully analyzed the calculated complexes, taking into consideration their energies, number of hydrogen bonds and orientation of the ligand with respect to the Trp-286 aromatic rings. It was established by mutation [42] and NMR [43] experiments that this unique residue is important for ligand’s anchoring in the binding pocket and transcription of genes controlled by VDR.

**Figure 2:**
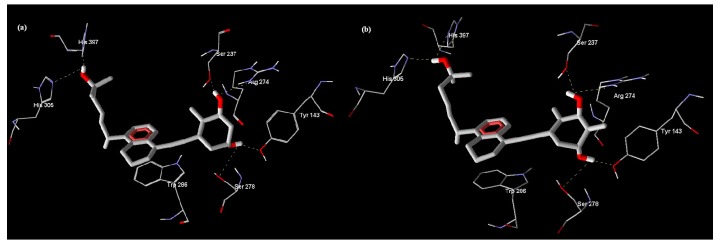
View of the three-dimensional structure of ligand binding cavity of the human vitamin D receptor (hVDR) with the docked (Molegro Virtual Docker) compounds **5** (**a**) and **6** (**b**). The six amino acids (Tyr 143, Ser 237, Arg 274, Ser 278, His 305 and His 397) forming hydrogen bonds with the ligand are depicted. The aromatic D ring of the ligands is parallel to the tryptophan molecule (Trp 286).

The docking studies show that both compounds **5** and **6** anchor the receptor similarly to calcitriol (Figure 2a,b). The notable difference consists in the “shift” of both ligands in the binding pocket [35], resulting in close proximity of their D ring to the parallel-oriented tryptophan rings. Such orientation of both aromatic fragments and the distance between them (ca. 4 Å) allows for their mutual π–π interaction [44,45]. Hydroxyl groups of the steroidal ligands create six hydrogen bonds with the same receptor residues as it was found in the crystalline complex hVDR-**1** [41].

### 2.3. Biological Evaluation: Binding to the Vitamin D Receptor

The affinities of the synthesized compounds **5** and **6** to VDR were assessed by a fluorescence polarization (FP)-based competition assay. The VDR affinities of compounds were checked using a wide range of concentrations and compared to that of calcitriol. Dose-response curves were plotted using GraphPad Prism software (version 6.04, GraphPad Software, Inc., San Diego, CA, USA), and half maximal inhibitory concentration (IC_50_) values were calculated from these dose-response curves. The binding affinities of compounds were compared to that of calcitriol, and they are presented in Table 1. Compound **5**, unsubstituted at C-2, was practically devoid of binding affinity to the VDR, whereas its analog **6** with the 2-exomethylene group was twenty times less potent than calcitriol.

### 2.4. Biological Evaluation: Differentiation of HL-60 Cells

HL60 cells were used to determine prodifferentiating activities of new analogs [46]. After initial screening, the concentration ranges were established for each compound. Compound **5** was tested at concentrations from 3 × 10^−8^ M to 10^−6^ M, compound **6** at concentrations from 6.25 × 10^−8^ M to 10^−6^ M, while calcitriol was applied at concentrations from 10^−10^ M to 10^−7^ M. The cells were exposed to the compounds for 96 h and then the expression of differentiation markers CD14 and CD11b was studied using flow cytometry. CD14 is an antigen characteristic for monocytes and macrophages [47], while CD11b is an integrin present on monocytes, macrophages and granulocytes [48]. Percentages of CD14- and CD11b-positive cells were read out using Becton Dickinson Accuri C6 software (Becton Dickinson, San Jose, CA, USA). Half maximal effective concentrations (EC_50_) values were estimated from dose-response curves plotted with GraphPad Prism software. Compound **5** was not active in inducing differentiation at all. Compound **6**, however less active than calcitriol, was able to significantly upregulate CD14 expression (Table 2) and CD11b (Table 3).

## 3. Materials and Methods

### 3.1. Chemistry

Melting points (uncorrected) were determined on a SMP10 Stuart Scientific capillary melting point apparatus (Sunnyvale, CA, USA). Optical rotations were measured in chloroform using a Perkin-Elmer model 343 polarimeter (Shelton, CT, USA) at 24 °C. Ultraviolet (UV) absorption spectra were obtained on a Shimadzu UV-1800 UV spectrophotometer (Kyoto, Japan) in absolute ethanol. Nuclear magnetic resonance spectra were recorded in CDCl_3_ solutions using Bruker AVANCE 300 MHz (Karlsruhe, Germany) and Bruker AVANCE 500 MHz instruments. Chemical shifts (δ) are reported in parts per million relative to (CH_3_)_4_Si (δ 0.00) or solvent signal as an internal standard. Signals in ^1^H NMR spectra are described using the following abbreviations: s—singlet, d—doublet, t—triplet, q—quartet, quint—quintet, sext—sextet, m—multiplet, br—broad, narr—narrow. High-resolution mass spectra were recorded on LCT time-of-flight(TOF) or Mass Quattro LC spectrometers using electrospray ionization (ESI) technique.

Reactions were usually carried out with magnetic stirring. All reactions involving moisture- or oxygen-sensitive compounds were carried out under dry argon atmosphere. Reaction temperatures refer to external bath temperatures. Tetrahydrofuran was distilled from Na/benzophenone; dichloromethane and toluene were distilled from P_2_O_5_, whereas pyridine, diisopropylamine, diethylamine and triethylamine were distilled from CaH_2_. The organic extracts were dried over anhydrous MgSO_4_, filtered and concentrated using a rotary evaporator at a water aspirator pressure (20–30 mmHg). Reactions were monitored by thin-layer chromatography (TLC) using aluminum-backed MERCK 60 silica gel plates (Darmstadt, Germany) (0.2 mm thickness). The chromatograms were visualized first with ultraviolet light (254 nm) and then by immersion in a cerium-molybdenum solution [10 g Ce(SO_4_)_2_ × 4 H_2_O, 25 g phosphomolybdic acid, 60 mL H_2_SO_4_ and 940 mL H_2_O] or *p*-anisaldehyde solution (5 mL H_2_SO_4_, 1.5 mL glacial HOAc, 3.7 mL *p*-anisaldehyde, 135 mL H_2_O) followed by heating. High-performance liquid chromatography (HPLC) purifications were performed on Waters Associates (Milford, MA, USA) liquid chromatograph equipped with a Model 486 tunable absorbance detector (Milford, MA, USA).

5-Oxo-5,6,7,8-tetrahydronaphthalene-1-carbonitrile (**12**). To a stirred solution of 5-bromotetralone (**11**; 6.00 g, 26.67 mmol) in *N,N-*dimethylformamide (DMF) (30 mL) was added Zn(CN)_2_ (5.77 g, 53.33 mmol) under argon. Then, Pd(Ph_3_)_4_ (0.31 g, 0.27 mmol) was added and the mixture was stirred and heated to 110 °C. After 5 h, the mixture was cooled to room temperature, brine was added and it was extracted with diethyl ether. The combined organic phases were dried (MgSO_4_) and concentrated. The residue was purified by flash chromatography over silica using hexane/ethyl acetate (98:2) to afford unreacted **11** (1.40 g) and the nitrile **12** (4.56 g, 64% based on recovered starting material) as a pale yellow oil. ^1^H NMR (300 MHz, CDCl_3_): δ 2.20 (2H, quint, *J* = 6.3 Hz, 7-H_2_), 2.72 (2H, dd, *J* = 7.4, 5.8 Hz, 6-H_2_), 3.20 (2H, t, *J* = 6.1 Hz, 8-H_2_), 7.44 (1H, t, *J* = 7.8 Hz, 3-H), 7.82 (1H, dd, *J* = 7.8, 1.4 Hz, 2-H), 8.27 (1H, dd, *J* = 7.8, 1.4 Hz, 4-H); ^13^C NMR (75 MHz, CDCl_3_): δ 196.2, 147.4, 137.1, 133.4, 131.6, 127.2, 116.9, 112.9, 38.5, 28.0, 22.2; HRMS (ESI) mass calcd for C_11_H_9_NONa (M^+^ + Na) 194.0582, measured 194.0573.

(5*R*)- and (5*S*)-Hydroxy-5,6,7,8-tetrahydronaphthalene-1-carbaldehyde (**13**). Diisobutylaluminum hydride (1M in methylene chloride; 37.3 mL, 37.3 mmol) was added to a stirred solution of the nitrile **12** (3.19 g, 18.64 mmol) in methylene chloride (220 mL) at −78 °C under argon. The mixture was stirred for 2 h, and it was quenched by the addition of cold ethyl acetate and 1M HCl. When the mixture reached room temperature, it was extracted with methylene chloride. The combined organic phases were washed with saturated NaHCO_3_ and brine, dried over anhydrous MgSO_4_ and concentrated. The residue was purified by flash chromatography over silica using hexane/ethyl acetate (95:5) to afford the aldehyde **13** (1.98 g, 60%) as a pale yellow oil. ^1^H NMR (300 MHz, CDCl_3_): δ 1.75–1.90 (2H, m, 7-H_2_), 1.94–2.07 (2H, m, 6-H_2_), 2.32 (1H, br s, OH), 3.04–3.32 (2H, m, 8-H_2_), 4.79 (1H, narr m, 5-H), 7.37 (1H, t, *J* = 7.6 Hz, 3-H), 7.72 (2H, d, *J* = 7.6 Hz, 2- and 4-H), 10.21 (1H, s, CHO); ^13^C NMR (75 MHz, CDCl_3_): δ 192.9, 140.3, 139.5, 134.6, 133.7, 132.6, 126.3, 68.1, 31.4, 26.2, 18.3; HRMS (ESI) mass calcd for C_11_H_12_O_2_Na (M^+^ + Na) 199.0735, measured 199.0737.

(5*R*)- and (5*S*)-(Triethylsilyl)oxy-5,6,7,8-tetrahydronaphthalene-1-carbaldehyde (**14**). To a stirred solution of hydroxy aldehyde **13** (1.98 g, 11.22 mmol) in anhydrous methylene chloride (210 mL) and 2,6-lutidine (3.9 mL, 33.66 mmol), triethylsilyl trifluoromethanesulfonate (3.0 mL, 13.27 mmol) was added at −78 °C. After 1 h, the reaction was quenched with saturated NaHCO_3_ and extracted with methylene chloride. The organic phase was dried (MgSO_4_) and concentrated. The residue was purified by column chromatography over silica using hexane/ethyl acetate (96:4) to give the protected compound **14** (3.25 g, 99%) as a colorless oil. ^1^H NMR (300 MHz, CDCl_3_): δ 0.71 (6H, q, *J* = 7.8 Hz, Si*CH_2_*CH_3_), 1.02 (9H, t, *J* = 7.8 Hz, SiCH_2_*CH_3_*), 1.71–1.85 (2H, m, 7-H_2_), 1.94–2.12 (2H, m, 6-H_2_), 3.11–3.30 (2H, m, 8-H_2_), 4.82 (1H, m, 5-H), 7.36 (1H, t, *J* = 7.6 Hz, 3-H), 7.66–7.71 (2H, m, 2- and 4-H), 10.24 (1H, s, CHO); ^13^C NMR (75 MHz, CDCl_3_): δ 193.0, 141.5, 139.4, 133.9, 133.6, 132.2, 126.0, 69.1, 32.0, 26.1, 19.0, 6.9, 5.1; HRMS (ESI) mass calcd for C_24_H_44_O_2_SiNa (M^+^ + Na) 313.1600, measured 313.1608.

(5′*R*,2*E*)- and (5′*S*,2*E*)-3-[(5′-(Triethylsilyl)oxy-5′,6′,7′,8′-tetrahydronaphthalen-1′-yl]-acrylic acid ethyl ester (**15**). Ethyl (triphenylphosphoranylidene)acetate (11.69 g, 33.59 mmol) was added at 0 °C to a solution of the aldehyde **14** (3.25 g, 11.20 mmol) in anhydrous methylene chloride (23 mL). The mixture was stirred for 24 h at room temperature under argon, and it was quenched with saturated NH_4_Cl. The mixture was extracted with methylene chloride, and the organic phase was dried (MgSO_4_) and concentrated. The residue was purified by column chromatography over silica using hexane/ethyl acetate (95:5) to afford an ester **15** (3.81 g, 94%) as a colorless oil. ^1^H NMR (300 MHz, CDCl_3_): δ 0.70 (6H, q, *J* = 7.4 Hz, Si*CH_2_*CH_3_), 1.01 (9H, t, *J* = 7.4 Hz, SiCH_2_*CH_3_*), 1.33 (3H, t, *J* = 7.1 Hz, COOCH_2_*CH_3_*), 1.74–1.85 (2H, m, 7′-H_2_), 1.90–2.12 (2H, m, 6′-H_2_), 2.74–2.94 (2H, m, 8′-H_2_), 4.26 (2H, q, *J* = 7.1 Hz, COOCH_2_CH_3_), 4.80 (1H, m, 5′-H), 7.20 (1H, t, *J* = 7.4 Hz, 3′-H), 7.41–7.47 (2H, m, 2′- and 4′-H), 7.96 (1H, d, *J* = 15.8 Hz, 3-H); ^13^C NMR (75 MHz, CDCl_3_): δ 167.0, 142.4, 140.7, 136.1, 133.1, 130.0, 126.0, 125.5, 119.7, 69.2, 60.4, 32.2, 26.4, 19.0, 14.3, 6.9, 5.1; HRMS (ESI) mass calcd for C_21_H_32_O_3_Na (M^+^ + Na) 383.2019, measured 383.2017.

(5′*R*,2*E*)- and (5′*S*,2*E*)-3-[5′-(Triethylsilyl)oxy-5′,6′,7′,8′-tetrahydronaphthalen-1′-yl]-prop-2-en-1-ol (**16**). Diisobutyl-aluminum hydride (1 M in methylene chloride; 21.2 mL, 21.2 mmol) was added to a solution of the ester **15** (3.81 g, 10.58 mmol) in methylene chloride (130 mL) at −78 °C under argon. The mixture was stirred for 2 h, and it was quenched by the addition of cold ethyl acetate and saturated NH_4_Cl. When the mixture reached room temperature, it was extracted with methylene chloride. The combined organic phases were dried (MgSO_4_) and concentrated. The residue was purified by flash chromatography over silica using hexane/ethyl acetate (96:4 ≥ 9:1) to afford allylic alcohol **16** (3.30 g, 98%) as a colorless oil. ^1^H NMR (300 MHz, CDCl_3_): δ 0.70 (6H, q, *J* = 7.4 Hz, Si*CH_2_*CH_3_), 1.01 (9H, t, *J* = 7.4 Hz, SiCH_2_*CH_3_*), 1.64–1.68 (2H, m, 7′-H_2_), 1.88–2.10 (2H, m, 6′-H_2_), 2.60–2.84 (2H, m, 8′-H_2_), 4.31 (2H, d, *J* = 5.3 Hz, 1-H_2_), 4.80 (1H, m, 5′-H), 6.18 (1H, dt, *J* = 15.7, 5.3 Hz, 2-H), 6.79 (1H, d, *J* = 15.7 Hz, 3-H), 7.16 (1H, t, *J* = 7.8 Hz, 3′-H), 7.31–7.35 (2H, m, 2′- and 4′-H); ^13^C NMR (75 MHz, CDCl_3_): δ 141.5, 138.5, 132.9, 132.8, 130.6, 127.9, 126.2, 123.6, 71.2, 62.4, 32.2, 26.5, 19.4, 6.9, 5.1; HRMS (ESI) mass calcd for C_19_H_34_O_2_SiNa (M^+^ + Na) 341.1913, measured 341.1915.

[(2′*R*,3′*R*,5′′*R*)- and 2′*R*,3′*R*,5′′*S*)-3′-[(5′′-(Triethylsilyl)oxy-5′′,6′′,7′′,8′′-tetrahydronaphthalen-1′′-yl]-oxiran-2′-yl]-methanol (**17**). To a stirred suspension of the activated, powdered molecular sieves 4Å (68 mg) in anhydrous methylene chloride (3.2 mL) and (-)-diisopropyl *D*-tartrate (17 μL, 0.12 mmol) was added titanium(IV) isopropoxide (20 μL, 0.07 mmol) at 0 °C. The mixture was cooled to −20 °C and solution of *tert*-butyl hydroperoxide (5.5 M in decane; 150 μL, 0.83 mmol) was slowly added. After 45 min, a solution of alcohol **16** (220 mg, 0.69 mmol) in anhydrous methylene chloride (2.1 mL) was transferred via cannula. The stirring was continued at −20 °C for 3 h, and then the reaction was quenched with brine and extracted with methylene chloride. The organic phase was dried (MgSO_4_) and concentrated. The residue was purified by column chromatography over silica using hexane/ethyl acetate (8:2) to give colorless, oily epoxide **17** (185 mg, 79%). ^1^H NMR (300 MHz, CDCl_3_): δ 0.71 (6H, q, *J* = 7.4 Hz, Si*CH_2_*CH_3_), 1.02 (9H, t, *J* =7.4 Hz, SiCH_2_*CH_3_*), 1.73–1.87 (2H, m, 7′′-H_2_), 1.90–2.14 (2H, m, 6′′-H_2_), 2.61-2.94 (2H, m, 8′′-H_2_), 3.03 (1H, dt, *J* = 3.7, 2.4 Hz, 2′-H), 3.81 (1H, ddd, *J* = 12.7, 3.7, 1.4 Hz, one of 1-H_2_), 3.98–4.06 (2H, m, 3′-H and one of 1-H_2_), 4.81 (1H, narr m, 5′′-H), 7.14 (1H, d, *J* = 7.4 Hz, 4′′-H), 7.19 (1H, t, *J* ~ 7.5 Hz, 3′′-H), 7.36 (1H, m, 2′′-H); ^13^C NMR (75 MHz, CDCl_3_): δ 134.6, 128.1, 127.6, 125.9, 123.2, 123.1, 70.5, 61.3, 60.4, 53.7, 32.4, 25.7, 19.2, 6.9, 5.1; HRMS (ESI) mass calcd for C_19_H_30_O_3_Na (M^+^ + Na) 357.1862, measured 357.1867.

(2*S*,3*S*,*5′R*)- and (2*S*,3*S*,*5′S*)-3-[(5′-(Triethylsilyl)oxy-5′,6′,7′,8′-tetrahydronaphthalen-1′-yl]-butane-1,2-diol (**18**). To a vigorously stirred suspension of CuCN (120 mg, 1.35 mmol) in anhydrous diethyl ether (3.6 mL) at −78 °C, a solution of methyllithium (1.6 M in diethyl ether; 1.12 mL, 1.80 mmol) was added. The mixture was stirred for 1 h and a solution of epoxide **17** (150 mg, 0.45 mmol) in anhydrous diethyl ether (1.8 mL) was transferred via cannula. The cooling bath was removed and the mixture was allowed to reach 0 °C during 3 h. Then, it was quenched with saturated NH_4_Cl and extracted with ethyl acetate. The organic phase was dried (MgSO_4_) and evaporated. The residue was purified by column chromatography over silica using hexane/ethyl acetate (9:1 ≥ 8:2) to give colorless, oily diol **18** (127 mg, 80%). ^1^H NMR (300 MHz, CDCl_3_): δ 0.71 (6H, q, *J* = 7.4 Hz, Si*CH_2_*CH_3_), 1.02 (9H, t, *J* = 7.4 Hz, SiCH_2_*CH_3_*), 1.16 and 1.19 (1.5H and 1.5H, each d, *J* = 6.9 Hz, 4-H_3_), 1.67–1.84 (2H, m, 7′-H_2_), 1.91–2.10 (2H, m, 6′-H_2_), 2.63–2.91 (2H, m, 8′-H_2_), 3.22 (1H, m, 3-H), 3.60 (1H, dd, *J* = 11.2, 6.3 Hz, one of 1-H_2_), 3.82 (2H, narr m, 2-H and one of 1-H_2_), 4.81 (1H, narr m, 5′-H), 7.14–7.24 (2H, m, 2′- and 4′-H), 7.33 (1H, t, *J* = 7.6 Hz, 3′-H); ^13^C NMR (75 MHz, CDCl_3_): δ 141.2, 140.6, 139.5, 135.8, 135.6, 126.7, 126.3, 126.2, 124.7, 69.8, 69.7, 64.5, 36.3, 32.3, 29.7, 26.2, 19.4, 19.3, 17.9, 6.9, 5.1 Hz; HRMS (ESI) mass calcd for C_11_H_18_O_2_Na (M^+^ + Na) 373.2175, measured 373.2175.

(2*S*,5′*R*)- and (2*S*,5′*S*)-2-[5′-(Triethylsilyl)oxy-5′,6′,7′,8′-tetrahydronaphthalen-1′-yl]propionaldehyde (**19**). Sodium periodate (2.71 g, 12.65 mmol) and saturated NaHCO_3_ (1.3 mL) were added to a solution of the diol **18** (738 mg, 2.11 mmol) in anhydrous methylene chloride (10.5 mL). The mixture was vigorously stirred at room temperature for 1 h under argon. Then, reaction was diluted with saturated NaHCO_3_ and extracted with methylene chloride. The organic phase was dried (MgSO_4_) and evaporated. The residue was purified by column chromatography over silica using hexane/ethyl acetate (95:5) to afford aldehyde **19** (634 mg, 94%) as a colorless oil. ^1^H NMR (300 MHz, CDCl_3_): δ 0.71 (6H, q, *J* = 7.4 Hz, Si*CH_2_*CH_3_), 1.02 (9H, t, *J* = 7.4 Hz, SiCH_2_*CH_3_*), 1.37 (3H, d, *J* = 7.0 Hz, 3-H_3_), 1.73–1.86 (2H, m, 7′-H_2_), 1.93–2.14 (2H, m, 6′-H_2_), 2.62–2.87 (2H, m, 8′-H_2_), 3.82 (1H, dq, *J* = 1.2, 7.0 Hz, 2-H), 4.83 (1H, t, *J* = 5.5 Hz, 5′-H), 6.94 and 6.95 (0.5H and 0.5H, each d, *J* = 7.5 Hz, 2′-H), 7.23 (1H, t, *J* = 7.5 Hz, 3′-H), 7.38 and 7.40 (0.5H and 0.5H, each d, *J* = 7.5 Hz, 4′-H), 9.59 and 9.62 (0.5H and 0.5H, each d, *J* = 1.2 Hz, 1-H); ^13^C NMR (75 MHz, CDCl_3_): δ 201.2, 201.1, 141.1, 136.0, 135.4, 127.8, 127.6, 126.4, 126.4, 126.3, 69.5, 69.4, 48.6, 48.5, 32.3, 32.2, 26.3, 26.2, 19.4, 19.1, 14.5, 6.9, 5.1; HRMS (ESI) mass calcd for C_19_H_30_O_2_Na (M^+^ + Na) 341.1913, measured 341.1907.

(2*S*,5′*R*)- and (2*S*,5′*S*)-2-[5′-(Triethylsilyl)oxy-5′,6′,7′,8′-tetrahydronaphthalen-1′-yl]-propan-1-ol (**20**). Sodium borohydride (290 mg, 0.91 mmol) was added to a solution of the aldehyde **19** (52 mg, 1.37 mmol) in methanol (11.5 mL) at 0 °C. The cooling bath was removed and the mixture was stirred under argon at room temperature for 1 h. The solvent was evaporated and the residue was dissolved in ethyl acetate and extracted with brine. The organic phase was dried (MgSO_4_) and evaporated. The residue was purified by column chromatography over silica using hexane/ethyl acetate (8:2) to afford alcohol **20** (265 mg, 90%) as a colorless oil. ^1^H NMR (300 MHz, CDCl_3_): δ 0.73 (6H, q, *J* = 7.5 Hz, Si*CH_2_*CH_3_), 1.04 (9H, t, *J* = 7.5 Hz, SiCH_2_*CH_3_*), 1.22 and 1.25 (1.5H and 1.5H, each d, *J* = 6.9 Hz, 3-H_3_), 2.67–2.93 (2H, m, 8′-H_2_), 3.28 (1H, sext, *J* = 6.9 Hz, 2-H), 3.59–3.81 (2H, m, 1-H_2_), 4.85 (1H, m, 5′-H), 7.14 (1H, m, 2′-H), 7.23 and 7.24 (0.5H and 0.5H, each t, *J* = 7.6 Hz, 3′-H), 7.35 (1H, m, 4′-H); ^13^C NMR (75 MHz, CDCl_3_): δ 141.3, 140.4, 140.3, 135.2, 135 1, 126.5, 126.0 125.9, 124.2, 69.6, 68.1, 36.3, 36.3, 32.4, 26.0, 25.9, 19.4, 19.3, 17.8, 6.9, 5.2; HRMS (ESI) mass calcd for C_19_H_32_O_2_Na (M^+^ + Na) 343.2069, measured 343.2072.

Toluene-4-sulfonic acid [(2′*S*,5′′*R*)- and (2′*S*,5′′*S*)′-2′-[5′′-(triethylsilyl)oxy-5′′,6′′,7′′,8′′-tetrahydronaphthalen-1′′-yl]-propyl ester (**21**). To a stirred solution of alcohol **20** (100 mg, 0.31 mmol) in anhydrous methylene chloride (3 mL), triethylamine (103 μL, 1.41 mmol) and 4-(dimethylamino)pyridine (DMAP) (8 mg, 0.063 mmol), *p*-toluenesulfonyl chloride (89 mg, 0.469 mmol) was added at 0 °C. The reaction mixture was allowed to warm to room temperature and stirring was continued for 2 h. Methylene chloride (20 mL) was added and the organic phase was washed with saturated NaHCO_3_, dried (MgSO_4_) and concentrated under reduced pressure. The residue was purified by column chromatography over silica using hexane/ethyl acetate (9:1 ≥ 8:2) to afford tosylate **21** (136 mg, 91%) as a colorless oil. ^1^H NMR (300 MHz, CDCl_3_): δ 0.696 and 0.689 (3H and 3H, each q, *J* = 7.8 Hz, 3 × Si*CH_2_*CH_3_), 1.00 and 1.01 (4.5H and 4.5H, each t, *J* = 7.8 Hz, 3 × SiCH_2_*CH_3_*), 1.21 and 1.25 (1.5H and 1.5H, each d, *J* = 6.9 Hz, 3′-H_3_), 1.62–1.79 and 1.87–2.03 (2H and 2H, each m, 6”-H_2_ and 7”-H_2_), 2.43 (3H, br s, 4-CH_3_), 2.47–2.74 (2H, m, 8′′-H_2_), 3.33 (1H, sext, *J* = 6.9 Hz, 2′-H), 3.89–4.08 (2H, m, 1′-H_2_), 4.77 (1H, narr m, 5”-H), 6.94 (1H, dm, *J* = 7.5 Hz, 2”-H), 7.11 (1H, t, *J* = 7.5 Hz, 3”-H), 7.27–7.33 (3H, m, 4′′-H_2_, 3-H and 5-H), 7.66 and 7.69 (1H and 1H, each d, *J* = 6.5 Hz, 2-H and 6-H); ^13^C NMR (75 MHz, CDCl_3_): δ 144.6, 140.5, 140.3, 139.3, 134.6, 129.8, 129.7, 127.9, 127.0, 126.0, 124.4, 74.6, 69.5, 33.4, 32.2, 26.0, 21.6, 19.2, 19.1, 17.6, 6.9, 5.1; HRMS (ESI) mass calcd for C_26_H_37_O_4_SSiNa (M^+^ + Na) 496.2079, measured 496.2082.

(1*R*,1′*R*)- and (1*S*,1′*R*)-5-[1′,5′-Dimethyl-5′-(triethylsilyl)oxy-hexyl)]-1-(triethylsilyl)oxy-1,2,3,4-tetra-hydronaphthalene (**22**).

4-chloro-2-methyl-2-[(triethylsilyl)oxy]butane (**A**). To a stirred solution of 3-methyl-1,3-diol (182 mg, 1.75 mmol) in anhydrous pyridine (2.3 mL) was added *p*-toluenesulfonyl chloride (499 mg, 2.63 mmol) at 0 °C. The mixture was stirred for 2 h, and then it was poured into ice-cooled 2 M hydrochloric acid and extracted with ethyl acetate. The combined organic phases were washed with saturated NaHCO_3_ and brine, dried (MgSO_4_) and concentrated. The residue was purified by column chromatography over silica using hexane/ethyl acetate (8:2) to afford toluene-4-sulfonic acid 3-hydroxy-3-methyl-butyl ester (450 mg, 99%) as a colorless oil.

To a stirred solution of this tosylate (1.00 g, 3.88 mmol) in anhydrous methylene chloride (40 mL), imidazole (0.501 g, 7.36 mmol) and triethylsilyl chloride (0.97 mL, 5.81 mmol) were added at 0 °C. After 1 h, the reaction was quenched with saturated NH_4_Cl and extracted with methylene chloride. The organic phase was dried (MgSO_4_), and concentrated. The residue was purified by column chromatography over silica using hexane/ethyl acetate (96:4) to give toluene-4-sulfonic acid 3-[(triethylsilyl)oxy]-3-methyl-butyl ester (1.44 g, 99%) as a colorless oil.

To a solution of the silylated tosylate (4.00 g, 10.75 mmol) in DMF (60 mL), LiCl (2.26 g, 53.76 mmol) was added and the mixture was heated at 80 °C for 24 h with stirring. The solvent was evaporated, the water was added to the residue and the mixture was extracted with ethyl acetate. The combined organic phases were dried (MgSO_4_) and concentrated. The residue was purified by column chromatography over silica using hexane/diethyl ether (96:4) to give the chloro compound **A** (1.48 g, 58%) as a colorless oil. ^1^H NMR (300 MHz, CDCl_3_): δ 0.57 (6H, q, *J* = 7.5 Hz, 3 × Si-*CH_2_*-CH_3_), 0.94 (9H, t, *J* = 7.5 Hz, 3 × Si-CH_2_-*CH_3_*), 1.24 (6H, s, 2 × CH_3_), 1.93 (2H, m, Cl-CH_2_-*CH_2_*-), 3.63 (2H, m, Cl-*CH_2_*-CH_2_-).

The chloro compound **A** (244 mg, 1.04 mmol) was added dropwise to a vigorously stirred mixture of magnesium turnings (1.30 g) in anhydrous THF (4 mL) under argon. The reaction mixture was heated to reflux and second portion of chloride **A** (488 mg, 2.08 mmol) was added dropwise via a reflux condenser. After 10 min, a third portion of chloride **A** (488 mg, 2.08 mmol) was added and the mixture was refluxed for 30 min. The resulted solution of the Grignard reagent was diluted with THF (10 mL), cooled to −40 °C, and transferred to a cooled solution (−40 °C) of the tosylate 21 (615 mg, 1.30 mmol) in anhydrous THF (16 mL). After 15 min, a solution of CuI (0.986 g, 5.19 mmol) in anhydrous THF (8 mL) was added. The reaction mixture was stirred at −40 °C for 4 h. Then, it was quenched by addition of saturated NH_4_Cl and extracted with ethyl acetate. The combined organic phases were dried (MgSO_4_) and concentrated. The residue was purified by flash chromatography over silica using hexane/diethyl ether (98:2) to afford diether **22** (476 mg, 72%) as a colorless oil. ^1^H NMR (300 MHz, CDCl_3_): δ 0.51–0.63 (6H, m, Si*CH_2_*CH_3_), 0.73 (9H, q, *J* = 7.5 Hz, SiCH_2_*CH_3_*), 0.95 (6H, t, *J* = 7.5 Hz, Si*CH_2_*CH_3_), 0.98 (3H, d, *J* = 6.9 Hz, 1′-CH_3_), 1.05 (9H, t, *J* = 7.5 Hz, SiCH_2_*CH_3_*), 1.18 (6H, s, 5′-CH_3_ and 6′-H_3_), 2.68–2.83 (2H, m, 4-H_2_), 2.95 (1H, sext, *J* = 6.9 Hz, 1′-H), 4.82 (1H, t, *J* = 5.7 Hz, 1-H), 7.10 (1H, d, *J* = 7.5 Hz, 6-H), 7.17 and 7.18 (0.5H and 0.5H, each t, *J* = 7.5 Hz, 7-H), 7.26 and 7.28 (0.5H and 0.5H, each d, *J* = 7.5 Hz, 8-H); ^13^C NMR (75 MHz, CDCl_3_): δ 145.8, 145.7, 139.9, 139.7, 133.9, 133.9, 130.9, 128.8, 125.7, 125.7, 125.6, 125.4, 124.1, 124.0, 73.4, 73.4, 69.9, 69.8, 45.2, 45.1, 32.5, 29.9, 29.8, 29.7, 25.8, 22.7, 22.6, 19.4, 7.1, 6.9, 5.2; HRMS (ESI) mass calcd for C_30_H_56_O_2_Si_2_Na (M^+^ + Na) 527.3717, measured 527.3721.

(1*R*,1′*R*)- and (1*S*,1′*R*)-5-[1′,5′-Dimethyl-5′-(triethylsilyl)oxy-hexyl]-1,2,3,4-tetrahydronaphthalen-1-ol (**23**). To a solution of diether **22** (476 mg, 0.94 mmol) in THF (47 mL), tetrabutylammonium fluoride (1.0 M in THF; 18.9 mL, 18.9 mmol) was added at room temperature under argon. The stirring was continued for 30 min, brine was added, and the mixture was extracted with ethyl acetate. The organic extracts were dried (MgSO_4_) and evaporated. The residue was purified by column chromatography over silica using hexane/ethyl acetate (9:1 ≥ 1:1) to afford a protected alcohol **23** (345 mg, 93%) as a colorless oil. ^1^H NMR (300 MHz, CDCl_3_): δ 0.52 (6H, q, *J* = 7.5 Hz, Si*CH_2_*CH_3_), 0.90 (9H, t, *J* = 7.5 Hz, SiCH_2_CH_3_), 1.15 (6H, s, 5′-CH_3_ and 6′-H_3_), 1.25 (3H, d, *J* = 6.9 Hz, 1′-CH_3_), 2.98 (1H, sext, *J* = 6.9 Hz, 1′-H), 4.79 (1H, t, *J* = 5.7 Hz, 1-H), 7.15 (1H, dd, *J* = 7.7, 1.5 Hz, 6-H), 7.20 (1H, t, *J* = 7.7 Hz, 7-H), 7.93 (1H, m, 8-H); ^13^C NMR (75 MHz, CDCl_3_): δ 146.3, 138.9, 134.2, 126.4, 126.3, 126.2, 124.8, 73.3, 68.8, 45.1, 38.5, 31.5, 29.9, 29.7, 26.0, 22.6, 21.6, 21.4, 18.7, 18.5, 7.1, 6.7; HRMS (ESI) mass calcd for C_24_H_42_O_2_SiNa (M^+^ + Na) 413.2852, measured 413.2850.

(1′*R*)-5-[1′,5′-Dimethyl-5′-(triethylsilyl)oxy-hexyl]-1,2,3,4-tetrahydronaphthalen-1-one (**24**). Dess-Martin periodinane (563 mg, 1.33 mmol) was added to a solution of alcohol **23** (0.345 g, 0.89 mmol) in anhydrous methylene chloride (35 mL). The mixture was stirred at room temperature for 1 h under argon. Then, reaction was quenched with saturated Na_2_S_2_O_3_ and saturated NaHCO_3_. The mixture was extracted with methylene chloride, and the organic phase was dried (MgSO_4_) and evaporated. The residue was purified by column chromatography over silica using hexane/ethyl acetate (8:2) to afford ketone **24** (237 mg, 69%) as a colorless oil. [α]^24^_D_ + 3.6° (*c* 1.23, CHCl_3_); ^1^H NMR (300 MHz, CDCl_3_): δ 0.51 (6H, q, *J* = 7.5 Hz, Si*CH_2_*CH_3_), 0.89 (9H, t, *J* = 7.5 Hz, SiCH_2_*CH_3_*), 1.14 (6H, s, 5′-CH_3_ and 6′-H_3_), 1.22 (3H, d, *J* = 6.9 Hz, 1′-CH_3_), 2.07–2.20 (2H, m, 3-H_2_), 2.55–2.71 (2H, m, 2-H_2_), 2.91–2.99 (2H, m, 4-H_2_), 3.05 (1H, sext, *J* = 6.9 Hz, 1′-H), 7.30 (1H, t, *J* = 7.7 Hz, 7-H), 7.42 (1H, dd, *J* = 7.7, 1.5 Hz, 6-H), 7.93 (1H, dd, *J* = 7.7, 1.5 Hz, 8-H); ^13^C NMR (75 MHz, CDCl_3_): δ 198.9, 145.9, 141.4, 132.9, 130.6, 126.4, 124.9, 73.2, 45.0, 38.6, 38.4, 33.9, 29.9, 29.8, 25.8, 22.8, 22.4, 21.6, 7.1, 6.7; HRMS (ESI) mass calcd for C_24_H_40_O_2_SiNa (M^+^ + Na) 411.2695, measured 411.2697.

Trifluoro-methanesulfonic acid (1′*R*)-5-[1′,5′-dimethyl-5′-(triethylsilyl)-oxy-hexyl]-3,4-dihydro-naphthalen-1-yl ester (**10**). To a solution of *i*-Pr_2_NH (69 μL, 0.49 mmol) in dry THF (1.5 mL) at −78 °C, *n*-BuLi (1.6 M solution in hexanes; 300 μL, 0.48 mmol) was dropwise added. The mixture was stirred at −78 °C for 1 h, and then a solution of the ketone **24** (116 mg, 0.30 mmol) in dry THF (0.9 mL) was added. The reaction mixture was stirred for 10 min, and a solution of *N*-phenyltrifluoromethanesulfonimide (160 mg, 0.45 mmol) in dry THF (0.9 mL) was transferred via cannula. The reaction mixture was stirred at −78 °C for 1 h and at room temperature for 30 min. Water was added and the mixture was extracted with hexane. The combined organic phases were dried (MgSO_4_) and evaporated. The residue was purified by column chromatography over silica using hexane/ethyl acetate (99.9:0.1) to afford enol derivative **10** (147 mg, 94%) as a colorless oil. [α]^24^_D_ + 12.8° (*c* 1.02 CHCl_3_); ^1^H NMR (300 MHz, CDCl_3_): δ 0.52 (6H, q, *J* = 7.5 Hz, Si*CH_2_*CH_3_), 0.90 (9H, t, *J* = 7.5 Hz, SiCH_2_*CH_3_*), 1.15 (6H, s, 5′-CH_3_ and 6′-H_3_), 1.22 (3H, d, *J* = 6.9 Hz, 1′-CH_3_), 2.48 (2H, m, 3-H_2_), 2.88 (2H, t, *J* = 8.1 Hz, 4-H_2_), 3.01 (1H, sext, *J* = 6.9 Hz, 1′-H), 6.02 (1H, t, *J* = 4.8 Hz, 2-H), 7.23 (3H, narr m, 6-, 7- and 8-H); ^13^C NMR (75 MHz, CDCl_3_): δ 146.9, 145.1, 133.5, 128.6, 127.0, 119.1, 117.0, 73.3, 45.1, 38.4, 34.3, 30.0, 29.9, 22.5, 22.4, 22.2, 21.4, 7.1, 6.8; HRMS (ESI) mass calcd for C_25_H_39_F_3_O_4_SSiNa (M^+^ + Na) 543.2188, measured 543.2182.

(3′*S*,5′*R*,1′′R)-4-[[3′,5′-Bis(*tert*-butyldimethylsilyl)oxy]-2′-methyl-cyclohex-1′-enylethynyl]-8-[1′′,5′′-dimethyl-5′′-(triethylsilyl)oxy-hexyl]-1,2-dihydronaphthalene (**25**). To a solution of enyne **8** (36 mg, 95.19 µmol) and triflate **10** (45 mg, 86.54 µmol) in anhydrous DMF (700 µL) were added CuI (1.6 mg, 8.65 µmol), (PPh_3_)_2_Pd(OAc)_2_ (2.6 mg, 3.46 µmol) and Et_2_NH (700 μL) at room temperature under argon. After 45 min, the mixture turned deep reddish-brown. Water was added and the mixture was extracted with hexane, dried (MgSO_4_) and concentrated. The residue was applied on a silica Sep-Pak cartridge (2 g) (Milford, MA, USA) and eluted with hexane to afford compound **25** (17 mg, 26%). ^1^H NMR (300 MHz, CDCl_3_): δ 0.09, 0.12 and 0.13 (6H, 3H and 3H, each s, 4 × Si*CH_3_*), 0.53 (6H, q, *J* = 7.5 Hz, SiCH_2_CH_3_), 0.91 (9H, s, Si-*t*-Bu), 0.92 (9H, t, *J* = 7.5 Hz, SiCH_2_*CH_3_*), 0.93 (9H, s, Si-*t*-Bu), 1.15 (6H, s, 5′′-CH_3_ and 6′′-H_3_), 1.22 (3H, d, *J* = 6.9 Hz, 1′′-CH_3_), 2.01 (3H, s, 2′-CH_3_), 2.38 (2H, m, 2-H_2_), 2.83 (2H, t, *J* = 7.9 Hz, 1-H_2_), 3.04 (1H, sext, *J* = 6.9 Hz, 1′′-H), 4.14 (1H, m, 5′β-H), 4.25 (1H, m, 3′α-H), 6.48 (1H, t, *J* = 4.9 Hz, 3-H), 7.16 (1H, dd, *J* = 7.6, 1.5 Hz, 7-H), 7.22 (1H, t, *J* = 7.6 Hz, 6-H), 7.51 (1H, dd, *J* = 7.6, 1.5 Hz, 5-H); HRMS (ESI) mass calcd for C_45_H_78_O_3_Si_3_Na (M^+^ + Na) 773.5157, measured 773.5165.

(3′*S*,5′*R*,1′′R)-4-[[3′,5′-Bis(*tert*-butyldimethylsilyl)oxy]-2′-methyl-4′-methylene-cyclohex-1′-enylethynyl]-8-[1′′,5′′-dimethyl-5′′-(triethylsilyl)oxy-hexyl]-1,2-dihydronaphthalene (**26**). To a solution of dienyne **9** (42 mg, 107.89 µmol) and triflate **10** (51 mg, 98.08 µmol) in anhydrous DMF (800 µL) were added CuI (1.8 mg, 9.81 µmol), (PPh_3_)_2_Pd(OAc)_2_ (2.9 mg, 3.92 µmol) and Et_2_NH (800 μL) at room temperature under argon. After 45 min, the mixture turned deep reddish-brown. Water was added and the mixture was extracted with hexane, dried (MgSO_4_) and concentrated. The residue was applied on a silica Sep-Pak cartridge (2 g) and eluted with hexane to afford compound **26** (17 mg, 23%). ^1^H NMR (300 MHz, CDCl_3_): δ 0.07, 0.08, 0.09 and 0.13 (each 3H, each s, SiCH_3_), 0.52 (6H, q, *J* = 7.5 Hz, Si*CH_2_*CH_3_), 0.89 (9H, s, Si-*t*-Bu), 0.91 (9H, t, *J* = 7.5 Hz, SiCH_2_*CH_3_*), 0.92 (9H, s, Si-*t*-Bu), 1.14 (6H, s, 5′′-CH_3_ and 6′′-H_3_), 1.20 (3H, d, *J* = 6.9 Hz, 1′′-CH_3_), 2.02 (3H, s, 2′-CH_3_), 2.37 (2H, 2-H_2_), 2.81 (2H, t, *J* = 7.9 Hz, 1-H_2_), 3.02 (1H, sext, *J* = 6.9 Hz, 1′′-H), 4.50–4.64 (2H, m, 3′α-H and 5′β-H), 4.95 and 5.18 (1H and 1H, each s, =CH_2_), 6.47 (1H, t, *J* = 4.9 Hz, 3-H), 7.15 (1H, dd, *J* = 7.6, 1.5 Hz, 7-H), 7.21 (1H, t, *J* = 7.6 Hz, 6-H), 7.48 (1H, dd, *J* = 7.6, 1.5 Hz, 5-H); HRMS (ESI) mass calcd for C_46_H_78_O_3_Si_3_Na (M^+^ + Na) 785.5157, measured 785.5153.

(1*R*,3*S*,1′′*R*)-5-[5′-(5′′-Hydroxy-1′′,5′′-dimethylhexyl)-3′,4′-dihydro-naphthalen-1′-ylethynyl]-4-methyl-cyclohex-4-ene-1,3-diol (**5**). To a solution of the protected compound **25** (17 mg, 22.66 µmol) in THF (1 mL) was added tetrabutylammonium fluoride (1.0 M in THF; 1.36 mL, 1.36 mmol) at room temperature under argon. The stirring was continued for 20 h, brine was added, and the mixture was extracted with ethyl acetate. The organic extracts were dried (MgSO_4_) and concentrated. The residue was applied on a silica Sep-Pak cartridge (2 g) and eluted with hexane/ethyl acetate (2:8) to afford triol **5** (8 mg, 85%). Further purification was achieved by HPLC (10 mm × 25 cm Luna Silica column, 4 mL/min) using hexane/2-propanol (8:2) solvent system; compound **5** was collected at R_V_ 71.5 mL. Analytical sample was obtained after reversed-phase HPLC (9.4 mm × 25 cm Zorbax Eclipse XDB-C18 column, 4 mL/min) using methanol/water (8:2) solvent system (R_V_ 50 mL). UV (in EtOH) λ_max_ 236, 251, 260 (ε 16900), 284 (br) nm; ^1^H NMR (500 MHz, CDCl_3_): δ 1.17 (6H, s, 5′′-CH_3_ and 6′′-H_3_), 1.21 (3H, d, *J* = 6.9 Hz, 1′′-CH_3_), 2.09 (3H, s, 4-CH_3_), 2.82 (2H, t, *J* = 8.2 Hz, 4′-H_2_), 4.18 (1H, m, 1β-H), 4.32 (1H, narr m, 3α-H), 6.49 (1H, t, *J* = 4.9 Hz, 2′-H), 7.16 (1H, dd, *J* = 7.9, 1.3 Hz, 6′-H), 7.21 (1H, t, *J* = 7.9 Hz, 7′-H), 7.48 (1H, dd, *J* = 7.9, 1.1 Hz, 8′-H); ^13^C NMR (75 MHz, CDCl_3_): δ 144.2, 140.2, 134.9, 132.7, 132.5, 126.3, 125.3, 123.1, 122.5, 116.0, 91.5, 89.4, 71.0, 69.4, 63.7, 44.0, 40.0, 39.2, 38.2, 29.3, 29.2, 25.7, 23.8, 22.6, 22.5, 21.6, 18.9; HRMS (ESI) mass calcd for C_27_H_36_O_3_Na (M^+^ + Na) 431.2562, measured 431.2561.

(1*R*,3*S*,1′′*R*)-5-[5′-(5′′-Hydroxy-1′′,5′′-dimethylhexyl)-3′,4′-dihydro-naphthalen-1′-ylethynyl]-4-methyl-2-methylene-cyclohex-4-ene-1,3-diol (**6**). To a solution of the protected compound **26** (17 mg, 22.31 µmol) in THF (1 mL) was added tetrabutylammonium fluoride (1.0 M in THF; 1.33 mL, 1.33 mmol) at room temperature under argon. The stirring was continued for 20 h, brine was added and the mixture was extracted with ethyl acetate. The organic extracts were dried (MgSO_4_) and concentrated. The residue was applied on a silica Sep-Pak cartridge (2 g) and eluted with hexane/ethyl acetate (2:8) to afford compound **6** (8 mg, 85%). Further purification was achieved by HPLC (10 mm × 25 cm Luna Silica column, 4 mL/min) using hexane/2-propanol (8:2) solvent system (R_V_ 37 mL); analytical sample was obtained after reversed-phase HPLC (9.4 mm × 25 cm Zorbax Eclipse XDB-C18 column, 4 mL/min) using methanol/water (8:2) solvent system (R_V_ 61 mL). UV (in EtOH) λ_max_ 236, 252, 261 (ε 17,000), 285 nm; ^1^H NMR (500 MHz, CDCl_3_): δ 1.17 (6H, s, 5′′-CH_3_ and 6′′-H_3_), 1.21 (3H, d, *J* = 6.9 Hz, 1′′-CH_3_), 2.12 (3H, s, 4-CH_3_), 2.82 (2H, t, *J* = 7.9 Hz, 4′-H_2_), 4.69 (2H, m, 1β- and 3α-H), 5.18 and 5.23 (1H and 1H, each s, =CH_2_), 6.49 (1H, t, *J* = 4.9 Hz, 2′-H), 7.16 (1H, dd, *J* = 7.9, 1.3 Hz, 6′-H), 7.21 (1H, t, *J* = 7.9 Hz, 7′-H), 7.47 (1H, dd, *J* = 7.9, 1.1 Hz, 8′-H); ^13^C NMR (75 MHz, CDCl_3_): δ 149.9, 144.2, 140.0, 135.1, 132.6, 132.5, 126.3, 125.3, 123.1, 122.6, 116.5, 108.9, 91.8, 89.0, 73.7, 67.4, 44.0, 40.7, 39.2, 38.3, 29.3, 29.2, 25.6, 23.8, 22.6, 22.5, 21.6, 18.8; HRMS (ESI) mass calcd for C_28_H_36_O_3_Na (M^+^ + Na) 443.2562, measured 443.2566.

### 3.2. Biological in Vitro Studies

Binding affinity to VDR was evaluated using a PolarScreen^TM^ Vitamin D Receptor Competitor Assay Kit under the manufacturer’s conditions (Invitrogen, Carlsbad, CA, USA). All compounds were evaluated within the concentration range of 10^−13^–10^−5^ M; the concentrations were determined using UV spectrophotometry. In this assay, recombinant human VDR was added to a fluorescent VDR ligand to form a complex, resulting in a high fluorescence polarization value. Then, the tested compounds were added to the complex into 386-well plates. The tested compounds were incubated for 4 h at room temperature in order to reach equilibrium. They displaced the fluorescent ligand from the complex, resulting in lower polarization value. The polarized fluorescence of every plate was measured three times using Envision multiplate reader (PerkinElmer, Waltham, MA, USA) and mean fluorescence polarization was calculated from these measurements. The whole assay was repeated in triplicate. IC_50_ values were calculated in GraphPad Prism (version 6.04, GraphPad Software, San Diego, CA, USA) using the average of values obtained.

HL60 cells were obtained from the Institute of Immunology and Experimental Therapy (Wrocław, Poland). The cells were cultured at standard cell culture conditions. The cells were seeded at a density of 15 × 10^4^ cells/mL in culture medium containing calcitriol, an analog or the equivalent volume of ethanol as a vehicle control. After 96 h of incubation, the cells were washed in phosphate buffered saline (PBS) incubated for 1 h on ice with 1 µL CD14-PE and 1 µL CD11b-FITC (both ImmunoTools, Friesoythe, Germany). Cells were washed and suspended in 350 µL of PBS prior to analysis on the Becton Dickinson Accuri C6 (San Jose, CA, USA). Data analysis was performed using Becton Dickinson Accuri C6 software. The assay was repeated from three (compound **5**) to five (calcitriol and compound **6**) times. Percentages of positive cells were plotted to the graphs and EC_50_ values were calculated using GraphPad Prism software.

### 3.3. Molecular Modeling and Docking to the VDR

The molecular mechanics studies were used to establish the energy-minimized conformations of the synthesized compounds **5** and **6**. The calculation of optimized geometries was initially carried out using the algorithm from the MM+ HyperChem (release 8.0) software package (Hypercube, Inc., Gainesville, FL, USA). MM+ is an all-atom force field based on the MM2 functional form. The procedure used for finding the global minimum structures was analogous to that described previously by us for the vitamin D side chain conformers [49] and involved the Conformational Search module. The calculated global minimum conformers were next energy-minimized using PCModel (release 9.0) program (Serena Software, Bloomington, IN, USA). Then, they were docked into the ligand binding pocket of the vitamin D receptor, extracted from crystalline hVDR (LBD)-**1** complex (Protein Data Bank, Code: 1DB1), using Molegro Virtual Docker (release 4.0) program (CLC bio, Qiagen, Aarhus, Denmark).

## 4. Conclusions

Two compounds, characterized by a presence of dienyne moiety conjugated with the aromatic D ring, were successfully synthesized using convergent strategy. Despite the polyunsaturated nature of their structures, these compounds, contrary to the classical vitamin D analogs, cannot undergo undesired thermal isomerization to previtamin D forms. Compound with a 2-exomethylene substituent exhibited moderate affinity to the VDR predicted by molecular docking experiments. The reason of drastically lower binding activity of its counterpart unsubstituted at C-2 is not clear, but this fact remains in agreement with the literature data indicating that a presence of such A-ring methylene moiety can significantly increase the VDR affinity of calcitriol analogs [34,50]. One possible explanation of this effect comes from the examination of crystal structures of the VDR bound to differently C(2)-substituted (2α-methyl, -ethyl, -propyl, etc.) vitamin D analogs. It was found that a 2α-methyl substituent, present in the VDR superagonists characterized by high binding affinity, provides additional van der Waals contacts, while being small enough not to destroy the water network present in the channel located near C-2 [51]. It can be, therefore, possible that the 2-methylene moiety exerts an analogous effect, interacting with the receptor in a similar manner. The new class of compounds, presented in this work, can be further modified and optimized in a search for potential VDR ligands exhibiting selective biological activities.

## Figures and Tables

**Table 1:** Affinities of the compounds to recombinant vitamin D receptor ^a^.

	Calcitriol	5	6
**IC_50_ (nM)**	1.42	ND	28.01

^a^ The VDR binding affinity is expressed as IC_50_. ND: not detected.

**Table 2:** Induction of CD14 expression by calcitriol and analogs ^a^.

	Calcitriol	5	6
**EC_50_ (nM)**	0.732	ND	155.5

^a^ EC_50_ values were estimated from dose-response curves using GraphPad Prism software. ND: not detected.

**Table 3:** Induction of CD11b expression by calcitriol and analogs **^a^**.

	Calcitriol	5	6
**EC_50_ (nM)**	9.163	ND	409.5

^a^ EC_50_ values were estimated from dose-response curves using GraphPad Prism software. ND: not detected.
